# Compounding of Liquid and Solid Dose Adjustable Formulations with Pantoprazole: Comparison of Stability, Applicability and Suitability

**DOI:** 10.3390/pharmaceutics15030717

**Published:** 2023-02-21

**Authors:** Nemanja Todorović, Jelena Čanji Panić, Mina Zavišić, Jelena Krtolica, Radomir Ratajac, Jelena Petrović, Dušica Bosiljčić, Nebojša Kladar, Nataša Milošević, Mladena Lalić-Popović

**Affiliations:** 1 Department of Pharmacy, Faculty of Medicine Novi Sad, University of Novi Sad, 21000 Novi Sad, Serbia; 2 Department of Drug Analysis and Veterinary Toxicology, Scientific Veterinary Institute Novi Sad, University of Novi Sad, 21000 Novi Sad, Serbia; 3 Galenic Laboratory, Pharmacy Elixa, 21000 Novi Sad, Serbia; 4 Centre for Medical and Pharmaceutical Investigations and Quality Control (CEMPhIC), Faculty of Medicine Novi Sad, University of Novi Sad, 21000 Novi Sad, Serbia

**Keywords:** syrups, capsules, dissolution, pediatric use, drug compounding, extemporaneous preparations, galenics, formulations

## Abstract

Pantoprazole is a model substance that requires dosage form adjustments to meet the needs of all patients. Pediatric pantoprazole formulations in Serbia are mostly compounded as capsules (divided powders), while in Western Europe liquid formulations are more common. The aim of this work was to examine and compare the characteristics of compounded liquid and solid dosage forms of pantoprazole. Three syrup bases were used: a sugar-free vehicle for oral solution (according to USP43-NF38), a vehicle with glucose and hydroxypropyl cellulose (according to the DAC/NRF2018) and a commercially available SyrSpend Alka base. Lactose monohydrate, microcrystalline cellulose and a commercially available capsule filler (excipient II, composition: pregelatinized corn starch, magnesium stearate, micronized silicon dioxide, micronized talc) were used as diluents in the capsule formulations. Pantoprazole concentration was determined by the usage of the HPLC method. Pharmaceutical technological procedures and microbiological stability measurements were performed according to the recommendations of the EP10. Although dose appropriate compounding with pantoprazole is suitable using both liquid vehicles as well as solid formulations, chemical stability is enhanced in solid formulation. Nevertheless, according to our results, if liquid formulation is a pH adjusted syrup, it could be safely kept in a refrigerator for up to 4 weeks. Additionally, liquid formulations could be readily applied, while solid formulation should be mixed with appropriate vehicles with higher pH values.

## 1. Introduction

Drug compounding in galenic laboratories and hospital or public pharmacies is often required in order to adjust the dosage and the formulation according to the specific needs of the patients. However, compounding with chemically instable active pharmaceutical ingredients (APIs) is challenging and literature data about the stability, the specific recommendation for preparation of product or the handling of such a product are scarce. Proton pump inhibitors (PPIs) are recognized as chemically instable APIs. They are sensitive to acidic environments, light, temperature, oxidative conditions and the presence of other salts. PPIs are inactive compounds that have to be activated by protonation (often falsely marked as prodrugs) in the acidic environment of parietal cells. Thus, PPIs must be protected against premature activation in the stomach by adequate formulation, such as enterocoated tablets, pellets and capsules. They are relatively stable at pH = 7.0, but decompose in acidic solutions, and gastroresistant coating provides side site-specific release in the small intestine [1,2,3,4,5].

Pantoprazole belongs to the benzimidazole PPI group that has an extended rate of metabolism resulting in a shorter lifetime in the plasma. Pantoprazole sodium sesquihydrate (PSS) is official according to the European Pharmacopoeia 10 (EP10) and it is classified as freely soluble in water (1–10 mL for 1 g PSS). Furthermore, it has been provisionally classified as a biopharmaceutics classification system (BCS) class III drug (i.e., high solubility and low permeability), and it is proposed as eligible for biowaiver. Pantoprazole is a liposoluble molecule (logP 2.11), that is a weak base (pKa 3.55 and 9.15) and it is unstable in acidic environments like the other PPIs [6,7,8,9,10].

Gastric acid secretion is suppressed by pantoprazole as a result of its covalent binding to cysteine residues in the α-subunit of H^+^/K^+^-ATPase [11,12]. Various diseases, such as erosive esophagitis associated with gastroesophageal reflux disease (GERD) and pathological hypersecretory conditions, including Zollinger–Ellison syndrome, are treated with pantoprazole [13]. In addition, pantoprazole is applied for ulcer prophylaxis in patients on multidrug therapy [13,14,15]. Furthermore, combined with antibiotics, pantoprazole has off-label usage in eradication of *Helicobacter pylori* [15]. Pantoprazole is metabolized by CYP2C19 with interindividual variations attributed to age and other medications [16], as well as gene polymorphism. The Food and Drug Administration (FDA) recognized pantoprazole as a drug with CYP2C19-variable metabolism, especially in pediatric populations that are acknowledged as poor pantoprazole metabolizers [17]. It was also reported that 57% variability in pantoprazole clearance among adults could be attributed to CYP2C19 genotype differences [18].

Pantoprazole is registered in Serbia, as in many other countries in the world, as an enteric coated solid dosage form (either a delayed-release capsule or tablet) or as an intravenous solution (a product for reconstitution) [19]. Pantoprazole solid dosage forms should not be broken; however, in certain cases such as in pediatric or geriatric populations, when dose adjustment is required, the solid dosage forms are applied after breaking. Thus, an easy to swallow formulation should be developed, which is preferred to intravenous formulation. Although multiparticle formulations of pantoprazole such as gastroresistant pellets or capsules filled with granules are registered, in many countries including Serbia there are only tablets or intravenous formulations on the market [19].

Taking into consideration its chemical characteristics and interindividual variability, as well as the lack of registered dosage forms that are age appropriate or easily adjustable for personalized therapy, pantoprazole is an eligible substance for drug compounding that requires knowledge of trained pharmacists. Facilities, time and expertise in pharmacies limit the type of compounded dosage forms that are usually prepared in practice. Many formulations are developed in-house, based on the literature available (if any), and there are few data regarding the harmonization of the formulations [20,21]. The quality of the formulations is usually evaluated indirectly; limited facilities are available for quality assurance with a lack of analytical equipment required for the uniformity evaluation of the drug content or its stability [22]. An advantage of extemporaneous liquid preparation is the possibility to omit preservatives which could cause side effects, especially in pediatric populations. Even though liquid medications are the preferred form for pediatric populations, they usually contain sugar that increases glycemic index, and if applied chronically, they could provoke dental caries. Furthermore, liquid formulations that contain citric acid could have an erosive effect when applied in the long term [23]. On the other hand, solid dosage form compounding, especially for APIs that are unstable in aqueous environments, have better drug stability, although only a few results of stability studies have been published for compounded capsules [22,23,24]. Additionally, solid dosage forms may be desirable because fewer excipients are needed [25]. Liquid extemporaneous forms are more common in North America and Western European countries, whereas powders are often prepared in Eastern Europe [26].

In this study, we mimicked the compounding of liquid and solid dosage forms in pharmacy. For the first time, three liquid pantoprazole formulations (one sugar free, one with glucose and one commercially available) and three powder pantoprazole formulations (using three common fillers: lactose, microcrystalline cellulose and commercially available filler) were investigated. Additionally, the impact of the syrup base type and the presence of preservative in various media on the pantoprazole chemical stability in liquid formulations was documented. The influence of dosing medium on the pantoprazole dissolution profile and its stability was also studied.

## 2. Materials and Methods

### 2.1. Materials

Enteric coated tablet formulations (Nolpaza^®^ 40 mg, Krka Farma doo, Novo Mesto, Slovenia) containing pantoprazole sodium sesquihydrate (PSS) were purchased from the local pharmacy. The substances used for the preparation of syrup bases were: hydroxyethyl cellulose (HEC, Sigma Aldrich, St. Louis, MO, USA), xanthan gum (Alekpharm doo, Belgrade, Serbia), glycerol (Lach:ner, Neratovice, Czech Republic), sorbitol (Carl Roth, Karlsruhe, Germany), sodium saccharin tablets (Instantina Ges.m.b.H, Dürnkrut, Austria), glucose (Sigma Aldrich, St. Louis, MO, USA), potassium sorbate (Alfa Aesar GmbH & Co, KG, Kandel, Germany), methyl paraben (Alfa Aesar GmbH & Co, KG, Kandel, Germany), citric acid (Sigma Aldrich, St. Louis, MO, USA), sodium citrate (Sigma Aldrich, St. Louis, MO, USA), sodium bicarbonate (Sigma Aldrich, St. Louis, MO, USA), SyrSpend Alka^®^ base (Fagron, Rotterdam, The Netherlands).

The following excipients were applied for the powder formulation: microcrystalline cellulose (MCC, Vivapur^®^ 101, JRS Pharma, Rosenberg, Germany), alpha-lactose monohydrate (LAC, Capsulac^®^, Meggle, Wasserburg am Inn, Germany); excipient II (EXCII, Farmalabor, Canosa di Puglia, Italy; composition: pregelatinized corn starch, magnesium stearate, micronized silicon dioxide, micronized talc). White-green hard gelatin capsules (size 0, Farmalabor, Canosa di Puglia, Italy) were used. Capsule shell composition: indigo (FD&C Blue 2), titanium dioxide, yellow iron oxide, gelatin.

Dissolution media were prepared in accordance with the regulations of the United States Pharmacopeia and the National Formulary (USP43-NF38) [27]. Concentrated hydrochloric acid (37%, PanReac, p.a., Barcelona, Spain) was diluted to obtain medium of pH 1.2 (0.1 M). Medium of pH 6.8 (phosphate buffer, 0.1 M) was composed using potassium dihydrogen phosphate (Lachner, p.a., Neratovice, Czech Republic) and sodium hydroxide (PanReac, p.a., Barcelona, Spain). Commercially available apple juice (Nectar, 100% fruit, Bačka Palanka, Serbia) and chamomile tea (Josif Pančić, Belgrade, Serbia) were used as dosing media. The selection was based on choosing local producers known for high quality of products and widespread usage by people in Serbia. Chamomile tea was prepared according to the traditional recommendation based on the monograph of the European Medical Agency (EMA) in the form of an infusion by pouring 150 mL of boiling water onto 1 g of chamomile flowers, filtered through cheesecloth and cooled before use [28].

Concentration of pantoprazole in syrup formulations and content of pantoprazole in stability studies were determined using the HPLC method. The mobile phase for HPLC was made from acetonitrile (JTBaker, NJ, USA) and phosphate buffer. Methanol (J.T. Baker, NJ, USA) was used for sample preparation for the HPLC method.

As a reference standard to validate analytical methods (UV/Vis spectrophotometry and HPLC), pure PSS (Lek Pharmaceuticals d.d, Ljubljana, Slovenia) was used. Specificity of the HPLC method was tested using standards of sodium citrate, potassium sorbate, methyl paraben, sorbitol, glucose and a solution of sodium saccharin tablets. Specificity of the UV/Vis method was tested using a solution of LAC, MCC, EXCII as well as apple juice and chamomile tea.

### 2.2. Compounding of Syrup Formulations

Three syrup formulations with PSS were formulated using three syrup vehicles, respectively. In each compounded liquid formulation, the pantoprazole content was 2 mg/mL. First, pantoprazole tablets were triturated with a mortar and pestle (Supplementary Figure S1). A portion of each vehicle base was transferred to the mortar with pantoprazole and the rest of the vehicle was used to rinse the mortar and the pestle (up to 100 mL or up to 100 g, Table 1), as per the protocol of compounding suspension, and thus three different liquid formulations were prepared. The obtained formulations were suspensions due to the insoluble parts of the tablet formulations in the applied vehicles. All formulations were prepared in triplicate in a total volume of 100 mL (or g) and obtained samples were separated into three different bottles: the first underwent microbiological testing, the second was stored in controlled refrigeration (2–8 °C) and the third was stored at controlled room temperature measured by a hydrometer with a thermometer (HTC288ATH, HTC Instruments, India). Bottles that were refrigerated prior to testing were kept at room temperature.

Three vehicles were used for preparing the liquid compounded formulations of which two are official: one was sugar free with sorbitol (F1, Vehicles for oral solution, Sugar free, 795, USP43-NF38) [27] and the other contained glucose and HEC as thickening agent (F2, NRF S52, *Deutscher Arzneimittel-Codex/Neues Rezeptur-Formularium*, DAC/NRF2018) [29]. The third base was commercially available vehicle SyrSpend Alka^®^ (F3, Fagron). The pH values of F1, F2 and F3 syrup vehicles had pH values of 4.5, 6.5 and 9.3, respectively. Since pantoprazole is stable at a pH above 7, alterations were carried out in order to increase the pH value to 8.4 by addition of 8.4% sodium bicarbonate solution. Formulations in the original vehicle and the 8.4% sodium bicarbonate vehicle both with preservative (as per official prescription) and without were prepared, respectively. Formulation F1 contains citric buffer and potassium sorbate and methyl paraben as preservative, but when sodium bicarbonate is added as chemical stabilizer for pantoprazole, the pH rises and potassium sorbate is not effective, thus it is removed from formulations. Methyl paraben was used as preservative in all formulations with preservative. In addition, since all preservatives could have side effects, especially in a vulnerable population such as the pediatric one, we have prepared formulations without preservative. Compounded F3 was prepared with a specific amount of purified water. The content of the compounded liquid formulations is presented in Table 1.

### 2.3. Characterization of Compounded Syrup Formulations

#### 2.3.1. Determination of Pantoprazole Concentration, Physical Appearance and Measurements of pH Value

Pantoprazole concentration was determined after compounding and after 7 and 28 days for all formulations in triplicate (refrigerated and kept at room temperature). Concentration was measured using the HPLC method (described in the section Analytical Methods). Each formulation was shaken by hand for approximately 30 s, and an aliquot of 1 mL was transferred into a 50 mL flask. Methanol was added and the flask was well shaken, then the mixture was transferred into Eppendorf tubes and centrifuged for 15 min at 3500 rpm. The supernatant was filtered (0.45 µm) and transferred into vials for HPLC analyses. All samples of compounded formulations were prepared and measured 2 h after preparation. Methanol was used to prevent additional decline in the pantoprazole concentration (especially in acidic syrup bases).

According to the pharmaceutical preparations (*Pharmaceutica*) monograph in the EP10, an appearance test (e.g., size, shape and color) is recommended for extemporaneous preparations. Appearance was tested according to the recommended test in the pharmaceutical preparations monograph in EP10 [10] for compounded syrups by visual inspection of color. Testing was performed after compounding and after a 7- and 28-day period, respectively, for all formulations under refrigerated conditions and those kept at room temperature. The expected result is no color alteration during the tested period, i.e., the initial color of syrup suspension is assumed to remain the same if formulations are stable.

Values of pH were measured with a digital pH-meter (InoLab, pH 720, United Kingdom) in triplicate. Measurements were carried out after compounding and after a 7- and 28-day period, respectively, for all formulations (stored under refrigerated conditions and kept at room temperature).

#### 2.3.2. Microbiological Studies

Microbiological tests of the liquid formulations were performed on the day of compounding (0 time) and after 7 and 28 days, respectively. Formulations that had the highest pantoprazole concentrations (i.e., optimal chemical stability) were selected for this test. Comparison was carried out for the selected formulations with and without preservative. Microbiological tests were performed in triplicate according to the 2.6.12 and 2.6.13 monographs for non-sterile products in the EP10 [10]. The microbial count was considered to be the average number of colony-forming units (CFU) found in agar. Syrup formulations were considered microbiologically acceptable if the total aerobic microbial count (TAMC) was ≤100 CFU/g, the total combined yeast and mold count was ≤ 10 CFU/g and the absence of *Escherichia coli* was confirmed.

### 2.4. Compounding of Capsule Formulations

Three pantoprazole solid dosage compounded formulations were prepared with three different excipients (LAC, MCC and EXCII), by applying a mass-based method for low-dose capsules. For each of the three excipients used, a size 0 capsule capacity (C) was determined. The commercially available tablets were crushed (Supplementary Figure S1) with a mortar and pestle (Table S1). The mass of filler needed for compounding was calculated according to Equation (1).

(1) $$m_{filler}=N\times \left(C-m\times \frac{D}{D_{0}}\right)$$

where: *m_filler_* is mass of filler in grams needed for compounding, *C* is capsule capacity (0.51 g LAC; 0.36 g MCC and 0.46 g EXCII), *N* is number of capsules, *m* is mass of one crushed tablet (average 0.20 g), *D*_0_ is dose of API in crushed tablets (40 mg) and *D* is desirable dose of API in compounded capsules (5 mg) (Table 2).

Mixing with fillers was carried out in a powder mixer (Farmalabor, Italy). The capsules were filled manually using a capsule machine (Farmalabor, Italy). Mass of capsules varied depending on the filler used and we prepared 80 capsules out of 10 tablets (Table 2). Powder mixture was added to suitable bottles (50% volume should be empty in order to allow tumble mixing), time of mixing was 15 min and speed of mixer rotation was 75 rpm.

Samples were taken for initial analysis, and the remaining samples were stored and left in room temperature storage conditions for analysis after 28 days.

### 2.5. Characterization of Compounded Capsule Formulations

#### 2.5.1. Uniformity of Mass, Uniformity of Content and Assay

Uniformity of mass and uniformity of content tests were performed according to the requirements of the EP10 [10].

The mass of 20 randomly selected intact capsules was measured. After that, the filling of the capsules was carefully removed and the mass of the capsule shells was measured. It was observed whether the variation in individual mass of the contents in relation to the mean value was within the allowed percentage deviation (7.5% for the capsules compounded in this work).

The pantoprazole content of 10 randomly selected capsules after compounding and after 28 days of storage at room temperature was determined spectrophotometrically (described in the section Analytical Methods). It was checked whether the individual pantoprazole contents were within the permissible range according to test B in relation to the mean value.

The assay was performed by comparing the mean pantoprazole content value (from the uniformity of content test) with the theoretical value (5 mg). Conclusions on product quality were made based on EU regulatory requirements [30].

#### 2.5.2. Dissolution Testing

A dissolution tester (DT800, Erweka, Hessen, Germany) was used to investigate the release rate of pantoprazole from compounded capsules. An apparatus with baskets was used, at 100 rpm and at 37 ± 0.5 °C. Dissolution medium was phosphate buffer pH 6.8 in a volume of 500 mL (volume adapted to the pediatric population). In order to test the impact of dosing medium on the results of the dissolution test, 100 mL of apple juice (recommended administration method for authorized pantoprazole delayed-release oral suspension [31]) and 100 mL of chamomile tea (available medium with more basic pH) were added to the dissolution medium. Compounded capsule formulations are assumed to be used via liquid dosing media and for the pediatric population the acceptable dosing medium volume is 100 mL, which represents 20% of the capacity of dissolution volume. By addition of 20% of dosing liquid, we tested whether the dosing medium would significantly alter the dissolution profile in more biorelevant environment, and if so, whether it might adhere to guidelines in dosing medium recommendations.

The results were compared with those obtained after adding an additional 100 mL of phosphate buffer. Samples of 5 mL were taken at the following time points: 5 min, 15 min, 25 min, 35 min, 45 min and 60 min. The concentration of pantoprazole in the samples was measured spectrophotometrically (described in the Section 2.7).

### 2.6. Chemical Stability of Pantoprazole

Chemical stability was tested by measuring pantoprazole concentration in dissolution media (pH 6.8 and pH 1.2), dosing liquids (apple juice and chamomile tea), as well as sodium bicarbonate solution and citrate buffer (both of which are present in some of the investigated syrup formulations). Testing was performed for 2 h at predetermined time points, and the aim was to determine chemical stability in the dissolution medium as well as forced degradation rate in an acidic environment. Forced degradation stability testing was coupled with stability of pantoprazole in syrup formulations and capsules that was measured during a 28-day period [32,33,34]. Influence of capsule filler on pantoprazole stability after opening the capsule was tested in pH 1.2 medium, as the medium in which the lowest pantoprazole chemical stability is expected at 2 h.

Testing was performed with powder obtained from crushed tablets or capsules. Powder was transferred to tested media (100 mL) and, after predetermined time points, an aliquot of 0.5 mL was transferred and mixed with methanol. Methanol was used in order to obtain the exact concentration of pantoprazole in aqueous media at selected time points without an additional decline in the pantoprazole concentration. Pantoprazole concentration was determined using the HPLC method (described in the Analytical Methods) 5, 15, 30, 60 and 120 min after addition of the investigated medium. All samples after mixing with methanol were filtered through a 0.45 µm membrane filter (Agilent Technology, SAD). Degradation rate in an acidic environment (forced degradation) is expected to follow exponential decline (first-order kinetics) [4] according to Equation (2):

(2) $$A=A_{0}\times e^{-kt}$$

where: *A*—concentration at time *t* [µg/mL], *A*_0_—theoretical concentration at time 0, *k*—constant of degradation and *t*—time.

### 2.7. Analytical Methods

#### 2.7.1. UV/Vis Spectrophotometry

Content of pantoprazole in capsules and concentration of pantoprazole in dissolution samples were determined using a previously published UV/Vis spectrophotometry method [35]. Measurements were performed at a wavelength of 288 nm. Calibration was linear in the range 2.5–40 µg/mL. Calibration standards were prepared using phosphate buffer pH 6.8.

#### 2.7.2. High-Performance Liquid Chromatography (HPLC)

Concentration of pantoprazole in syrup formulations and content of pantoprazole in stability studies were determined using a previously published HPLC method [36]. Reversed phase chromatography was performed on a chromatograph (Series 1100, Agilent Technology, Santa Clara, CA, USA) applying isocratic elution with mobile phase consisting of acetonitrile and phosphate buffer (10 mM, pH 7 adjusted with 0.1 M sodium hydroxide) at 36:68 (*v*/*v*) using a C18 column (5 µm, 4.6 × 150 mm, Zorbax Eclips Plus). Flow rate was 0.8 mL/min and elution was performed at room temperature. Ten microliters of sample (preparation described in the previous section) was injected and analyzed at a 288 nm wavelength. Limit of quantification was 0.16 µg/mL, and limit of detection was 0.07 µg/mL. Retention time was 2.5 min for pantoprazole, and no other ingredient showed interference at this retention time.

### 2.8. Statistical Analyses

All statistical procedures were performed using SPSS (v26, IBM, Chicago, USA). Data were represented as mean value with standard deviation. A Q test was applied to all data to exclude measurement errors. Mean values of continuous data were compared using an ANOVA test. Dissolution data were compared using a model-independent approach (i.e., f1 difference factor and f2 similarity factor) [37]. Mean values were compared using ANOVA (post hoc Tukey), differences were considered significant if *p* < 0.05.

## 3. Results

### 3.1. Characterization of Compounded Syrup Formulations

Visual examination of the prepared syrup formulation revealed color changes in some formulations immediately after the preparation. Namely, formulations F1N3 and F1N4 were red 2 h after preparation. Interestingly, formulations F1K3 and F1K4, which, unlike formulations F1N3 and F1N4, contain preservative as the only difference, were colored orange 2 h after the compounding. These four formulations were prepared without the addition of sodium bicarbonate (8.4%). For F2 formulations, identical results were obtained, i.e., all formulations without sodium bicarbonate were pinkish (Supplementary Figure S2a). After a 7-day period, discoloration appeared in formulations kept at room temperature with sodium bicarbonate, i.e., F1N1 changed color from white to yellow, as well as F1K1 (formulation with preservative) which was darker (Supplementary Figure S2b,c). The same color change occurred in F2N2 and F2K2 formulations, for which, as in previous formulations, those with preservative were a slightly darker color. Additionally, formulations without sodium bicarbonate had a different color in those samples with preservative, but their color was lighter in comparison with formulations without preservative. No sign of color change was observed in any of the refrigerated formulations with added sodium bicarbonate. Only the formulation with the SyrSpend base (F3) remained white after 7 days for samples kept both at room temperature and in a refrigerated environment. After 28 days, color changes were similar to those after 7 days for all formulations (Supplementary Figure S2d,e).

**Figure 1:**
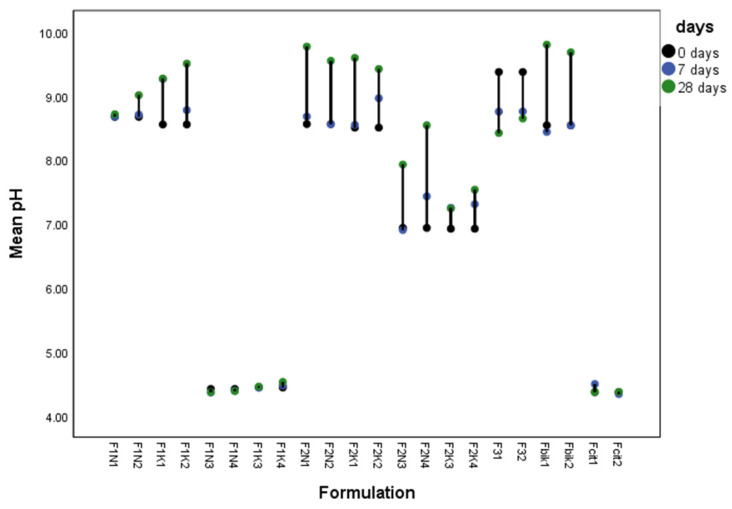
Bar chart of pH values in all investigated formulations (with and without preservative and refrigerated and those kept at room temperature) with addition of citrate buffer (0.16 M) and bicarbonate solution (8.4%) at 0, 7 and 28 days.

The initial pH values of the F1 formulations containing preservative with sodium bicarbonate were 8.67 ± 0.01 and 8.55 ± 0.02 in F1 formulations with preservative (i.e., F1N1/2 and F1K1/2, respectively), and 8.56 ± 0.01 in F2 and 8.50 ± 0.03 in F2 formulations with preservative (i.e., F2N1/2 and F2K1/2, respectively). In formulations without sodium bicarbonate, the initial pH in F1N3/4 formulations was 4.42 ± 0.02, in F1K3/4 it was 4.46 ± 0.03, in F2N3/4 it was 6.96 ± 0.02 and in F2K3/4 formulations it was 6.92 ± 0.03. In formulations with SyrSpend base, or F3 formulations, the initial pH was 9.37 ± 0.01 (Figure 1). In formulations without sodium bicarbonate at room temperature and refrigerated, the pH value remained unchanged after 7 and 28 days. In formulations with sodium bicarbonate with or without preservative, the pH slightly increased after 28 days. Formulations F2N1 and F2N2 had more pronounced pH changes than formulations F1N1 and F1N2 (Figure 1).

**Figure 2:**
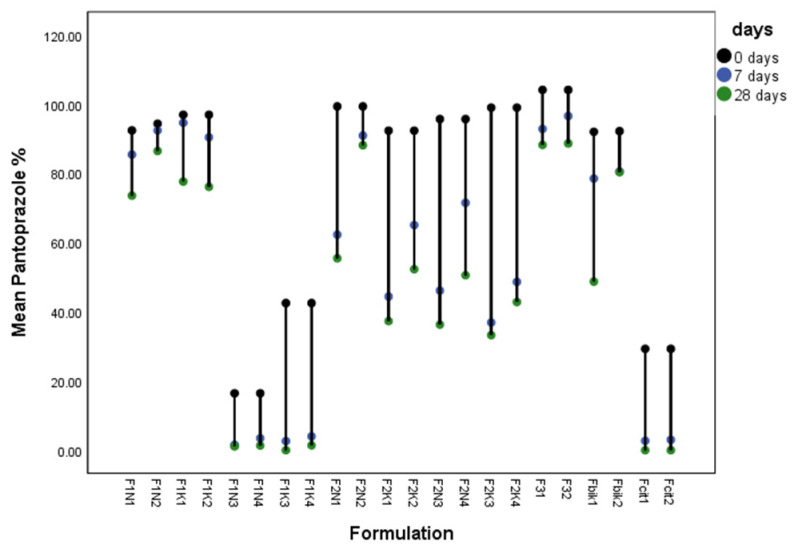
Content of pantoprazole (%) in 1 mL sample in all investigated formulations (with and without preservative and refrigerated and those kept at room temperature) with addition of citrate buffer (0.16 M) and bicarbonate solution (8.4%) at 0, 7 and 28 days.

Content of pantoprazole was influenced by pH, storage condition, syrup base and preservative (methyl paraben and potassium sorbate) (Figure 2). F1N2, F2N2 and F31 as well as F32 have same decline in concentration at 0 vs. 7 days and 7 vs. 28 days (Figure 2 and Supplementary Table S1), i.e., the concentrations on day 0 vs. 7 and day 0 vs. 28 are not statistically significantly different. Additionally, the concentrations of pantoprazole of F1N2, F2N2 and F31, F32 were compared among themselves at 0, 7 and 28 days, and there are no statistically significant differences (Supplementary Table S2). Formulations with preservative and sodium bicarbonate were more stable in F1 syrup base than in F2 syrup base. Formulation F1N2 had the same pantoprazole content from 0 to 28 days without statistically significant changes (Supplementary Table S1). Formulations without sodium bicarbonate had a significant concentration decline, and are not suitable for compounding. F1N1 and F1N2 have statistically the same concentrations at 0 and 7 days (p 0.974 and p 0.444), but after 28 days F1N1 had a statistically lower concentration (76 ± 2% of initial labeled concentration) and thus F1N2 (86 ± 4%) was selected as the more stable formulation (*p* < 0.045), which is presented in the Supplementary Materials (Table S1). Compared with the suspension of pantoprazole, all syrup formulations with added sodium bicarbonate were more stable (statistically significantly higher concentrations after 7 and 28 days, respectively).

We also tested whether official syrup base could be safely kept for 28 days without preservative. The results of microbiological stability testing of syrups indicated that all formulations complied with pharmacopeial specifications for liquid non-sterile products. The F2K2 formulation had slightly increased TAMC values, though it contained preservative, probably due to its crystallization in refrigerated conditions that could be seen during visual inspection (Table 3).

### 3.2. Characterization of Compounded Capsule Formulations

All capsule formulations complied with the uniformity of mass test (Table 4). Variations in mass of content were more pronounced in F4 formulations (with LAC as filler), in which mass content was the highest. The smallest variation in mass content was measured in F6 capsules (with EXCII as filler).

All capsule formulations compounded with three different excipients complied with the test of uniformity of content (Table 5). Similar lowest and highest contents were measured in F4 formulations. All average contents were in the range of 3.7–4.9% of theoretical drug content of 5 mg.

Uniformity of content was measured after 28 days and all formulations complied with the test, implying chemical stability of pantoprazole in capsule formulations (Table 6). There was no statistical difference in content variation in examined excipients. Percent of content compared to theoretical content (5 mg per capsule) was within EU requirements (±10%) and unchanged after 28 days in all three tested formulations (F1, F2, F3) as presented in Table 7.

**Figure 3:**
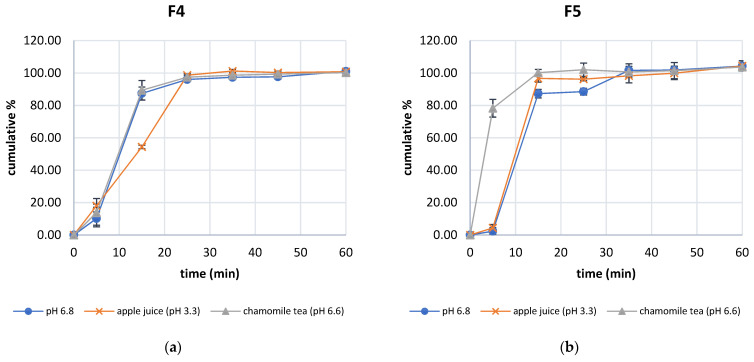

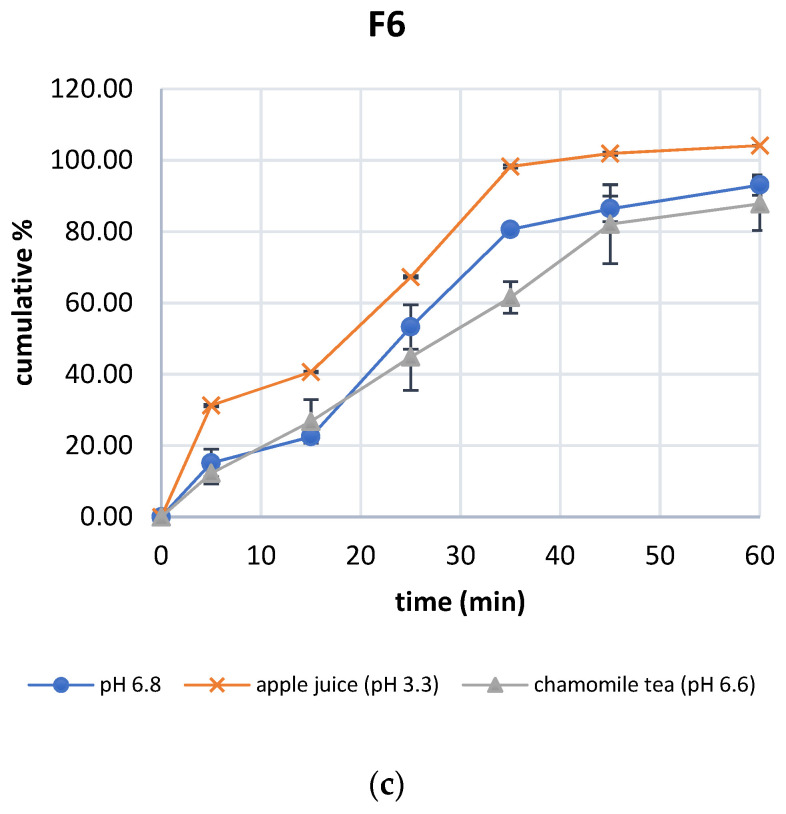
Dissolution profiles for pantoprazole compounded capsules in pH 6.8 dissolution medium and after the addition of 100 mL apple juice and chamomile tea, respectively, for (**a**) F4 (capsule with LAC), (**b**) F5 (capsule with MCC) and (**c**) F6 (capsule with EXCII).

All formulations complied with the requirement of pharmacopeia for conventional-release dosage forms and released more than 80% of the pantoprazole content after 45 min. In all three formulations, alteration of the dissolution profile was detected after addition of dosing liquids (Figure 3a–c). Dissolution profiles of F4 (with LAC) and F6 (with EXCII) after addition of 100 mL of apple juice were statistically different compared to the dissolution profile in pH 6.8 medium. Formulation F5 (with MCC) after addition of 100 mL of chamomile tea has a statistically different dissolution profile compared to the profile without the addition of the dosing media (selected as a reference formulation, Supplementary Table S3). Additionally, the release of pantoprazole from F6 was decreased with the addition of chamomile tea and increased with the addition of apple juice, which was not the case for the other excipients (Figure 3c).

Dissolution profile was also influenced by the excipients. The slowest release was measured in formulation F6 (with EXCII), which had peak release after 30 min. Profiles of all excipients were different in all three tested media (Supplementary Table S4).

### 3.3. Stability of Pantoprazole in Various Tested Media

**Figure 4:**
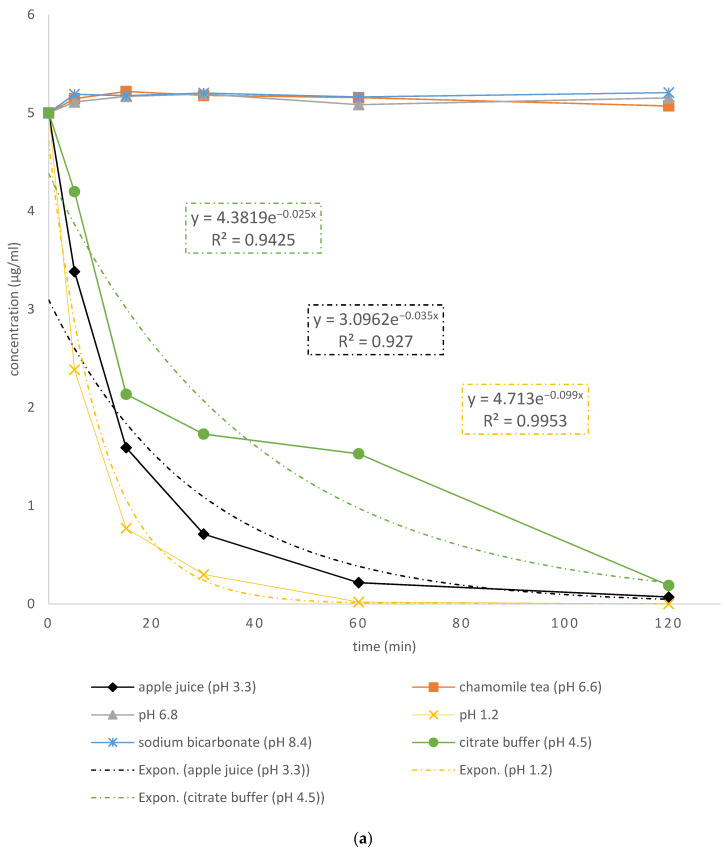

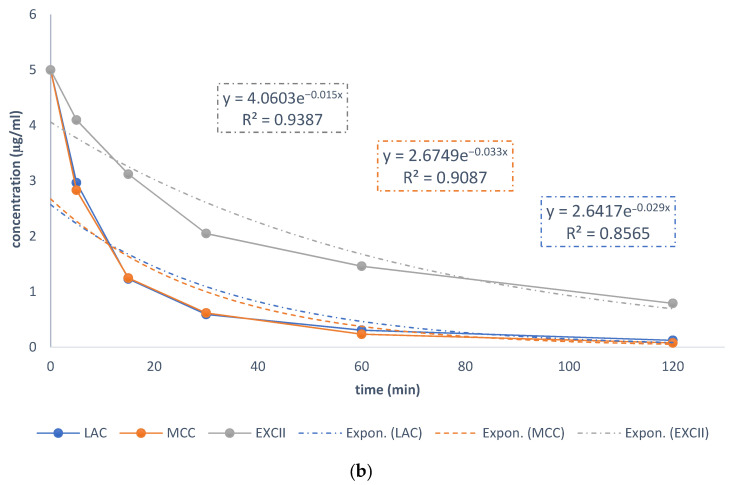
Kinetics of pantoprazole degradation in (**a**) tested dissolution media and dosing liquids; (**b**) medium pH 1.2 from compounded capsules F4, F5 and F6.

Figure 4a,b represent kinetics of pH-dependent pantoprazole degradation in dissolution media, liquid vehicles and dosing vehicles as well as impact of excipients on its degradation in acidic medium at pH 1.2. It is notable that pantoprazole is stable for two hours in dissolution medium at pH 6.8, chamomile tea (whose pH was 6.62) and sodium bicarbonate (whose pH was 8.5).

However, the pantoprazole degradation rate in an acidic environment, i.e., citric buffer (pH around 4.4), apple juice (pH 3.26) and in pH 1.2, was exponential. The lower the pH, the faster the pantoprazole degradation; thus, the degradation rate declines inversely to pH (slowest is in citric buffer and fastest in medium pH 1.2). Pantoprazole degradation half-life in pH 1.2 was around 5 min while in citric buffer and apple juice it was around 15 min (Figure 4a). Pantoprazole degradation half-life of F4 formulations (with LAC) was around 15 min while in F6 formulations (with EXCII) it was around 30 min, meaning degradation of pantoprazole is twice as slow in F6 capsules (Figure 4b). Kinetics of pantoprazole degradation in all acidic mediums best fitted to exponential decline (Figure 4a,b).

## 4. Discussion

The pharmaceutical industry, governments and healthcare professionals are encouraged to work together in order ensure that patients will receive high-quality standardized extemporaneously compounded non-sterile preparations (CNSPs). Pantoprazole is on the Food and Drug Administration (FDA) list as an API that should be the next generation to be developed as commercially available FDA-approved finished liquid dosage forms along with 16 other compounds [38]. However, there are also enteric coated pellet formulations with pantoprazole that are unavailable in many markets, thus compounding pantoprazole formulations that would be dose adjustable or easy to swallow is often a requirement.

In this study, three syrup formulations were compounded, using two officially available syrup bases: one that is sugar free and official in USP43-NF38 [27], or F1 formulation, and the other with glucose and official in DAC/NRF2018 [24], or F2 formulation. The third formulation (F3) contained the commercially available vehicle SyrSpend Alka^®^ (Fagron, the Netherlands). Pantoprazole, like many other PPIs, requires an alkaline ingredient with sufficient acid-neutralizing capacity to facilitate the drug’s safe passage through the stomach [4,5]; hence, 8.4% sodium bicarbonate solution was added. Previously, it was published that pantoprazole oral liquid formulation prepared extemporaneously from registered tablets in 8.4% sodium bicarbonate solution was stable in amber polyethylene terephthalate bottles for 62 days at 2–8 °C [39]. Additionally, it was reported that a crushed pantoprazole tablet after buffering with sodium hydrogen carbonate (bicarbonate) is bioequivalent to intact enteric coated pantoprazole in healthy fasting human subjects [32,40].

Visual appearance is pivotal for patients’ compliance. Even though the formulation is chemically stable, it becomes unacceptable for the patients if the color or the smell of the pharmaceutical form undergo changes. In this study, pantoprazole in all observed formulations was chemically unstable at a pH value below 7, as expected. Pantoprazole remained chemically stable in the formulations with pH value above 7, but those formulations stored at room temperature had an altered appearance. The findings in this study are in accordance with the data regarding omeprazole compounded liquid formulations. Namely, 2 mg/mL omeprazole in sodium bicarbonate solution was stable for 14 days at 24 °C and for as many as 30 days at 5 and −20 °C, respectively. In addition, liquid compounded omeprazole formulation stored at −20 and 5 °C had no color alterations during the 30-day study period. However, omeprazole liquid compounded formulation stored at 24 °C gradually changed from white to brown [41]. Variability in the pH value is crucial for the chemical stability of liquid pantoprazole formulations. The pH value remained unchanged in all liquid pantoprazole formulations observed in this study, except in the formulation with sodium bicarbonate stored in the refrigerator after 28 days. In a similar study conducted with omeprazole, the initial pH of the oral liquid formulation was 8.1 in each storage condition. The pH values increased over a 30-day period by 1.0, 0.6 and 0.5 pH units at 24, 5 and −20 °C, respectively [41]. In addition, all formulations had mild increases in pH values apart from F3 formulations for which the pH values slightly decreased. SyrSpend^®^ SF Alka contains calcium carbonate, maize starch and sucrose and was the syrup base in F3 formulations. Previously published studies reported the possibility of calcium carbonate precipitation in the presence of soluble starch [42], which might explain the decrease in pH value of F31 and F32 over time. In this research, the chemical stability of pantoprazole in the liquid compounded formulations was affected not only by the pH value of the syrup base but also by the formulation composition. Alterations in the official formulation by adding sodium bicarbonate led to chemically more stable pantoprazole syrups. The pantoprazole content in the liquid forms was influenced by the syrup base type. In the investigated formulations, the highest pantoprazole stability was assessed in the commercially available SyrSpend base (F3) after 7 and 28 days, respectively. Although the refrigerated pantoprazole liquid formulation F3 had pantoprazole content above 95% of the theoretical value after 7 days, the pantoprazole concentration declined to under 95% (88 ± 3.4%) after 28 days.

Pantoprazole formulation with sorbitol (F1) had higher stability after 7 and 28 days when compared to formulations with HEC (F2). In addition, the liquid pantoprazole formulations with added sodium bicarbonate (F1N1 and F2N2) were more stable in comparison with those without sodium bicarbonate, with even more pronounced differences in terms of stability when formulations F1 and F2 kept at room temperature were compared. In another study, omeprazole syrups with xanthan gum and sodium bicarbonate were stable over a 30-day period when stored under refrigerated conditions [33], confirming that the syrup base composition affects PPI chemical stability. According to our findings, pantoprazole liquid formulation F1N2 with preservative was more chemically stable in comparison with the F1K2 formulation without preservative (both formulations contain sodium bicarbonate). Additionally, the pantoprazole content of F1N2 on the 28th day remained unaltered when compared to the content on the first day. Both formulations F1N2 and F2N2 had the same pantoprazole content as determined in the commercial formulation F3. The content of pantoprazole in formulation F2N2 at 28 days was slightly higher than in formulation F1N2, both kept in a refrigerator, but a decreased content was more pronounced in formulation F2N2. Pantoprazole in sodium bicarbonate solution was stable as determined by previous research [36], but the addition of other excipients as in formulations F1 and F2 with added sodium bicarbonate resulted in improved pantoprazole stability. Increased pantoprazole stability in SyrSpend when compared to its stability in sodium bicarbonate solution was also reported [43].

The pantoprazole degradation rate increased with the pH value decrement. In this study, almost the entire amount of pantoprazole degraded at pH 4.5 (citrate buffer) within 2 h. Our results are in concordance with the pantoprazole degradation half-life at ambient temperature which was reported as approximately 2.8 h at pH 5.0 and approximately 220 h at pH 7.8 [44]. Pantoprazole formulations F2N3 (without preservative) and F2K3 (with preservative) with pH values 6.9 and 7.25, respectively, stored at room temperature reached half the pantoprazole content after 7 days, with a smaller amount of pantoprazole in F2K3 that contained preservative. The formulations F2N4 (without preservative) and F2K4 (with preservative) with pH values 6.9 and 7.25, respectively, kept in the refrigerator, had a significant decrease in pantoprazole. Both F2N4 and F2K4 contained half of the theoretical pantoprazole amount even after a 28-day period. The pantoprazole content in F2N4 was 70% after 7 days and was higher than the pantoprazole content quantified in the formulation with preservative, F2K4. A similar effect on concentration was detected in formulations stored at different temperatures, F1K3 and F1K4. The sorbic acid degradation depends on pH value, water activity, presence of other additives, conditions of storage and processing [45]. In addition, pantoprazole formulations F1K3 and F1K4 with sorbitol were orange colored while F2K3 and F2K4, that do not contain sorbitol, were pink, which could be the result of the extent of degradation in aqueous systems of sorbate to acetaldehyde and carboxyacrolein, and its rapid polymerization into brown pigments [46].

Lower pantoprazole concentrations were detected in formulations with preservative, F1K1 and F1K2, as well as F2K1 and F2K2, than those without methyl paraben (preservative). Pantoprazole concentration decline was more pronounced in formulations F2K1 and F2K2 in comparison with the sugar-free formulations with sorbitol (F1K1 and F1K2). In addition, pantoprazole concentration reduction was enhanced in formulations with methyl paraben (F1K1, F1K2, F2K1, F2K2) after 28 days in comparison to those without preservative (F1N1, F1N2, F2N1, F2N2). Methyl paraben, a preservative added in this study in the syrup base with sodium bicarbonate, is prone to degradation at pH above 5.5 and at higher temperatures, and the degradation could be additionally mitigated by other excipients in the formulation. Degradation of methyl parabens leads to production of p-hydroxybenzoic acid and methyl alcohol [47]. Pantoprazole’s interaction with the methyl paraben degradation products could result in lower pantoprazole concentration in alkaline formulations after 7 and 28 days, respectively.

In the study with omeprazole liquid formulations, no significant changes in omeprazole concentrations were reported after 30 days when stored at −20 or 5 °C, respectively. However, on day 18, the omeprazole concentration in the liquid form stored at 24 °C was only 86.3% of the initial omeprazole content [41].

Even though syrup formulation has better control of the medium, powder formulations have an advantage in terms of chemical stability. Moreover, when powder formulations are applied in a pediatric population, parents could choose the favorite beverage or meal to mix with the medication prior to application [48]. Due to several disadvantages of oral liquid medicines such as bad taste, portability problems or refrigerated storage conditions, the World Health Organization (WHO) currently prefers that young children, particularly in developing countries, are treated with oral solid medicines [49,50].

LAC and MCC were selected as excipients in this study as a common filler in compounded capsules since no interaction between pantoprazole and these excipients is expected [51]. However, there are no studies of the possible interaction of pantoprazole with EXCII. Since in this study, for the first time, certain advantages and altered dissolution behavior of EXCII combined with pantoprazole are reported, we believe that this study contributes knowledge about EXCII as a recommended filler in capsule compounding.

Uniformity of capsule mass as well as uniformity of pantoprazole content were in compliance with the pharmacopeial requirements. However, there were differences when different excipients were used in the formulation. A higher variation in content as well as in mass was measured in F4 capsules (with LAC). The smallest variation was observed when EXCII was used (F6 formulations), which was expected since it is designed as an excipient by the manufacturer of the equipment and capsules used in the compounding procedure in this research. The mass of capsule was highest in the F4 formulations (0.54 ± 0.02), then F6 (0.48 ± 0.00), and the smallest capsule mass was observed in F5 (0.37 ± 0.00). Additionally, assay testing confirms that the compounding technique using a capsule machine is suitable for obtaining compounded dosage forms with a low dose of API. All contents were within EU requirements [30].

The dissolution profile of compounded capsules was affected by the type of excipient and dissolution medium. The addition of apple juice altered the dissolution profile of the F4 formulation (with LAC) and F6 formulation (with EXCII): it decreased the dissolution rate in the F4 formulation but increased it in the F6 formulation. Formulation F6 had a different dissolution profile from F4 and F5 formulations in all investigated media. This is in correlation with stability studies where pantoprazole was the most stable in an acidic environment in F6 capsules (with EXCII). In addition, EXCII contains pregelatinized starch and magnesium stearate and thus pantoprazole’s dissolution release kinetics were the slowest with capsules compounded with this excipient. Although pantoprazole is usually formulated as enteric coated tablets, the addition of buffering agent might provide sufficient pantoprazole pH protection to retain its therapeutic efficacy [32,52]. Additionally, pulverized pantoprazole tablets with buffering agent had sharper pH elevation in comparison with intact tablets. Namely, the buffering agent added into powder formulation may provide sufficient pantoprazole protection [52].

Pantoprazole stability in crushed tablets in this study was tested in chamomile tea (pH 6.62), apple juice (pH 3.26) and sodium bicarbonate solution for 2 h. Pantoprazole was proven to be stable in sodium bicarbonate solution and chamomile tea for 2 h, which proves that these media are suitable as dosing vehicles. Exponential pantoprazole degradation was observed in apple juice and in pH 1.2 dissolution medium as well as in citric buffer. The decline in pantoprazole concentration in apple juice was two-fold slower than in pH 1.2 medium and almost three times slower in citric buffer, due to higher pH, which is in concordance with previously reported findings [53] that pantoprazole degradation was significantly pH dependent. Pantoprazole capsule F6 had a significantly slower pantoprazole degradation rate when compared to its degradation in F5 and F6 capsules, respectively. The decreased pantoprazole degradation rate in F6 capsules (with EXCII) could be attributed to the pregelatinized starch. At a lower pH, pregelatinized starch alters the physical properties, i.e., improves water absorption, viscosity and gel firmness [54]. In our study, pregelatinized starch offered a protective effect against pH-dependent pantoprazole degradation. However, these features of pregelatinized starch might contribute to the slower dissolution rate of pantoprazole from F6 capsules (compounded using EXCII).

## 5. Conclusions

The obtained results will be helpful for pharmacists in hospital and community pharmacies for preparing dose adjustable and easy to swallow compounded pantoprazole formulations, allowing the pharmacist to select the vehicle, the pH value, the preservative and the storage temperature for liquid formulations and the filler for solid formulations.

This study showed that storage at room temperature was not suitable for the liquid formulations. Chemical stability of pantoprazole was achieved by the addition of sodium bicarbonate in the official syrup bases investigated in this study. The presence of preservative had an effect on chemical stability as well as appearance. Additionally, better chemical stability was achieved in sugar-free formulations (F1). Formulations without preservative (with official syrup bases and commercial syrup base) kept in a refrigerator were microbiologically stable for 28 days. In conclusion, formulations compounded with official syrup bases, i.e., F1 (sugar-free formulation) and F2 (formulation with glucose and hydroxyethyl cellulose), with added sodium bicarbonate and without preservative, have the same chemical and microbiological stability as formulation F3 which was prepared using a commercially available syrup base.

Solid dosage forms of pantoprazole were prepared using commercial tablets and different vehicles/fillers. Capsule formulations have less complex content and compounding procedures and do not require special storage conditions. In this study, we have proved that dosing medium has an impact on dissolution profiles which makes this type of dissolution testing useful to health professionals in practice. Capsule formulations are best suited to be taken with a more basic dosage medium (such as chamomile tea). Acidic liquids such as apple juice are not suitable dosing media for compounded capsule formulations of pantoprazole.

## Figures and Tables

**Table 1:** Compositions of prepared sample suspensions with pantoprazole (2mg/mL) and different syrup vehicles.

Ingredients	F1 (USP43-NF38)								F2 (DAC/NRF2018)								F3 (SyrSpend)	
	pH 8.4				pH 4.5				pH 8.4				pH 6.5					
	Presence of Preservative																	
	no		yes		no		yes		no		yes		no		yes			
	Storage Temperature (°C)																	
	25	2–8	25	2–8	25	2–8	25	2–8	2–8	25	25	2–8	25	2–8	25	2–8	25	2–8
	Labels																	
	F1N1	F1N2	F1K1	F1K2	F1N3	F1N4	F1K3	F1K4	F2N1	F2N2	F2K1	F2K2	F2N3	F2N4	F2K3	F2K4	F3.1	F3.2
PSS	200 mg		200 mg		200 mg		200 mg		200 mg		200 mg		200 mg		200 mg		200 mg	
xanthan gum	0.05		0.05		0.05		0.05											
glycerol	10 mL		10 mL		10 mL		10 mL											
sorbitol	25 mL		25 mL		25 mL		25 mL											
saccharin	2 tbl		2 tbl		2 tbl		2 tbl											
HEC									0.5		0.5		0.5		0.5			
glucose									11.0		11.0		11.0		11.0			
potassium sorbate							0.1								0.14			
methyl paraben			0.1				0.1				0.1							
citric acid					1.5		1.5						0.07		0.07			
sodium citrate					2.0		2.0											
sodium bicarbonate	8.4		8.4						8.4		8.4							
SyrSpend base																	100 mL	
distilled water	up to 100 mL		up to 100 mL		up to 100 mL		up to 100 mL		up to 100 g		up to 100 g		up to 100 g		up to 100 g			

**Table 2:** Composition of compounded pantoprazole capsules for eight units.

Ingredient	F4	F5	F6
hard gelatin capsule (number)	8	8	8
tablet containing 40 mg pantoprazole * (number)	1	1	1
LAC (g)	4.88		
MCC (g)		2.68	
EXCPII ** (g)			3.48

* Excipients in tablet formulation: Mannitol (E421); Crospovidone; Sodium carbonate; Sorbitol (E420); Calcium stearate in tablet core and Hypromellose (E464); Povidone K25; Titanium dioxide (E171); Iron (III) oxide, yellow (E172); Propylene glycol (E1520); Methacrylic acid–ethacrylate copolymer (1:1), dispersion 30%; Sodium lauryl sulfate; Polysorbate 80; Macrogol 6000; Talc (E553b) in tablet film; ** composition: pregelatinized corn starch, magnesium stearate, micronized silicon dioxide, micronized talc.

**Table 3:** Microbiological test of selected syrup formulations (formulations with and without preservative, respectively) stored in refrigerated environment for 0, 7 and 28 days, respectively. Results are presented as colony-forming units (CFU)/g.

	F1N2			F1K2			F2N2			F2K2			F3.2		
days	0	7	28	0	7	28	0	7	28	0	7	28	0	7	28
Total aerobic microbial count (TAMC) *	10	10	40	<10	<10	<10	10	10	20	<10	20	40	<10	<10	<10
Total combined yeast and mold count (TYMC) **	<10	<10	<10	<10	<10	<10	<10	<10	<10	<10	<10	<10	<10	<10	<10
*Escherichia coli* ***	Abs	Abs	Abs	Abs	Abs	Abs	Abs	Abs	Abs	Abs	Abs	Abs	Abs	Abs	Abs

Acceptance criteria: * TAMC = 10^2^; ** TYMC = 10^1^; *** *E. coli* absent (Abs).

**Table 4:** Uniformity of mass test for three capsule formulations.

Sample	F4		F5		F6	
	m (g)	% *	m (g)	% *	m (g)	% *
	Mass		Mass		Mass	
	Content		Content		Content	
1	0.505	5.930	0.363	0.854	0.478	0.018
2	0.549	−2.325	0.367	−0.113	0.479	−0.087
3	0.558	−3.851	0.367	−0.252	0.477	0.319
4	0.545	−1.476	0.370	−1.181	0.478	−0.038
5	0.521	3.026	0.367	−0.107	0.475	0.645
6	0.556	−3.530	0.367	−0.129	0.479	−0.120
7	0.546	−1.716	0.367	−0.260	0.477	0.221
8	0.543	−1.202	0.369	−0.700	0.483	−0.917
9	0.554	−3.232	0.368	−0.443	0.478	0.005
10	0.535	0.420	0.364	0.485	0.484	−1.302
11	0.533	0.718	0.367	−0.293	0.486	−1.580
12	0.509	5.137	0.365	0.234	0.477	0.300
13	0.549	−2.215	0.361	1.313	0.476	0.428
14	0.520	3.060	0.357	2.468	0.473	1.091
15	0.536	0.236	0.374	−2.131	0.476	0.447
16	0.513	4.494	0.358	2.173	0.481	−0.576
17	0.538	−0.124	0.364	0.647	0.472	1.367
18	0.537	−0.084	0.372	−1.691	0.477	0.248
19	0.544	−1.362	0.372	−1.689	0.479	−0.078
20	0.547	−1.902	0.363	0.814	0.480	−0.392
aver	0.537		0.366		0.478	
min		−3.851		−2.131		−1.580
max		5.930		2.468		1.367

* % is deviation of mass of the content from average mass for each capsule formulation, acceptance criterion 7.5% variation.

**Table 5:** Uniformity of pantoprazole content (mg/capsule) in three formulations (F4, F5, F6) after compounding.

Uniformity of Content						
Sample	F4		F5		F6	
	m (mg)	% *	m (mg)	% *	m (mg)	% *
1	4.972	94.82	5.174	99.71	5.142	98.53
2	5.595	106.70	5.168	99.61	5.585	107.02
3	5.772	110.07	5.345	103.01	4.914	94.16
4	4.865	92.78	5.221	100.62	4.899	93.87
5	4.993	95.22	5.096	98.21	5.602	107.34
6	5.012	95.58	5.321	102.55	5.009	95.98
7	5.398	102.94	4.995	96.27	5.432	104.08
8	4.998	95.31	5.563	107.21	5.113	97.97
9	5.478	104.47	4.902	94.47	5.289	101.34
10	5.354	102.10	5.102	98.33	5.205	99.73
aver	5.244		5.189		5.219	
min		92.78		94.47		93.87
max		110.07		107.21		107.34

* Acceptance criterion 85–115% of the average content.

**Table 6:** Uniformity of pantoprazole content (mg/capsule) in three formulations (F4, F5, F6) after 28 days.

Uniformity of Content after 28 days						
Sample	F4		F5		F6	
	m (mg)	%	m (mg)	%	m (mg)	%
1	5.186	98.92	5.311	102.23	5.184	99.41
2	5.699	108.71	5.167	99.46	5.396	103.47
3	5.211	99.40	5.394	103.82	5.747	110.20
4	5.138	98.01	5.018	96.59	5.059	97.00
5	5.415	103.29	5.242	100.90	5.112	98.02
6	4.998	95.34	5.285	101.73	5.086	97.52
7	5.258	100.30	5.022	96.67	5.101	97.81
8	5.263	100.39	5.386	103.67	5.247	100.61
9	5.248	100.11	5.125	98.65	5.118	98.14
10	5.009	95.55	5.001	96.26	5.101	97.81
aver	5.242		5.195		5.215	
min		95.34		96.26		97.00
max		108.71		103.82		110.20

**Table 7:** Results of assay testing after compounding and after 28 days.

Formulations	Content (% of Theoretical Value)	
	After Compounding	After 28 Days
F4	104.88	104.84
F5	103.78	103.90
F6	104.38	104.30

## Data Availability

Not applicable.

## Supplementary Materials

The following supporting information can be downloaded at: https://www.mdpi.com/article/10.3390/pharmaceutics15030717/s1, Figure S1. Particle size distribution of crushed tablets (crushing method: mortar and pestle). Figure S2. Color of prepared pantoprazole formulations (a) 0 days, all formulations, (b) 7 days, formulations kept at room temperature, (c) 7 days, formulations kept in refrigerator, (d) 28 days, formulations kept at room temperature and (e) 28 days, formulations kept in refrigerator. Table S1. Comparison of pantoprazole content (%) in prepared formulations, 0 vs. 7 days and 0 vs. 28 days (n = 3). Table S2. Comparison of pantoprazole content (%) in prepared formulations F1N1, F2N2, F31 and F32 at 0, 7 and 28 days (Student’s *t*-test, *p* < 0.05 statistical significance, n = 3). Table S3. Dissolution profile comparison between F4, F5 and F6 in medium with pH 6.8 (reference formulations) and dissolution profiles in media after addition of apple juice and chamomile tea (test formulations) using model-independent method (n = 6). Table S4. Dissolution profile comparison between F4 (capsules with LAC as reference formulation) and F5/F6 (test formulations) in different dissolution media using model-independent method (n = 6).
