# Supplementary material for: Compounding of Liquid and Solid Dose Adjustable Formulations with Pantoprazole: Comparison of Stability, Applicability and Suitability
Source: Pharmaceutics. 2023 Feb 21;15(3):717. doi: 10.3390/pharmaceutics15030717 (PMC10058901; doi:10.3390/pharmaceutics15030717)
Supplement: Supplementary file 1 [file pharmaceutics-15-00717-s001.zip › pharmaceutics-2164978-supplementary.pdf]

pharmaceutics

SUPPLEMENTARY MATERIALS

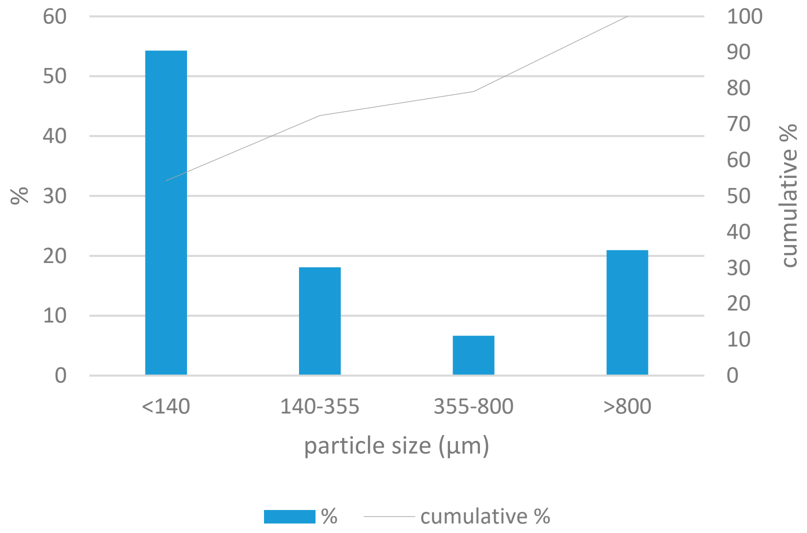

Figure S1. Particle size distribution of crashed tablets (crashing method mortar and pestle).

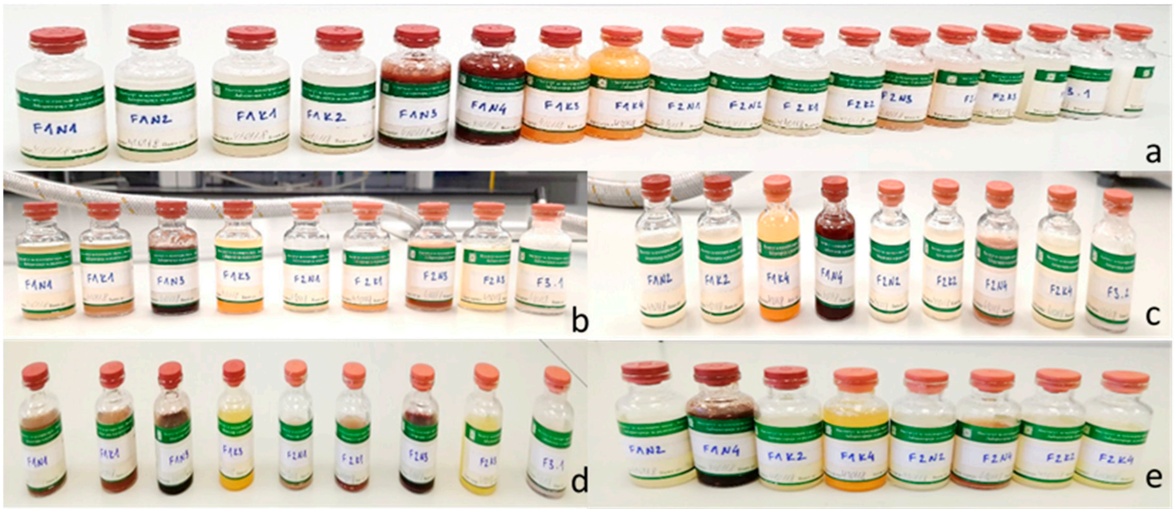

Figure S2. Color of prepared pantoprazole formulations a) 0 day all fromulations, b) 7 days fromulations kept on room temperature, c) 7 days formulations kept in refrigerator, d) 28 days formulations kept at room temperature and d) 28 days formulations kept in refrigerator

Table S1. Comparison of pantoprazole content (%) in prepared formulations 0 vs 7 days and 0 vs 28 days.

	**p values**	
**fromulation**	0 vs 7	0 vs 28
**F1N1**	0.020	0.000
**F1N2**	0.900	0.250
**F1K1**	0.797	0.004
**F1K2**	0.419	0.012
**F1N3**	0.000	0.000
**F1N4**	0.000	0.000
**F1K3**	0.000	0.000
**F1K4**	0.000	0.000
**F2N1**	0.000	0.000
**F2N2**	0.050	0.015
**F2K1**	0.000	0.000
**F2K2**	0.001	0.000
**F2N3**	0.000	0.000
**F2N4**	0.003	0.000
**F2K3**	0.000	0.000
**F2K4**	0.000	0.000
**F3.1**	0.720	0.180
**F3.2**	0.247	0.024

Table S2. Comparison of pantoprazole content (%) in prepared formulations F1N1, F2N2, F31 and F32 in 0, 7 and 28 days (Student T test, p<0.05 statistical significance).

**days**	**0**				**7**				**28**			
**fromulation**	**F1N2**	**F2N2**	**F31**	**F32**	**F1N2**	**F2N2**	**F31**	**F32**	**F1N2**	**F2N2**	**F31**	**F32**
**F1N2**	/	0.999	0.537	0.537	/	1.000	1.000	1.000	/	1.000	1.000	1.000
**F2N2**	0.999	/	0.999	0.999	1.000	/	1.000	0.998	1.000	/	1.000	1.000
**F31**	0.537	0.999	/	1.000	1.000	1.000	/	1.000	1.000	1.000	/	1.000

Table S3. Dissolution profiles comparison between F4, F5 and F6 in medium pH 6.8 (referent formulations) and dissolution profiles in mediums after addition apple juice and chamomile tea (test formulations) using model-independent method

**Excipients**	**Apple juice**		**Cmamomile tea**	
	**f1**	**f2**	**f1**	**f2**
F4	11	43	3	75
F5	6	63	23	26
F6	26	42	14	51

*Profiles are similar if f1 < 15 and f2 is between 50 and 100.

Table S4. Dissolution profiles comparison between F4 (capsules with LAC as referent formulation) and F5/F6 (test formulations) in different dissolution mediums using model-independent method

**Dissolution medium**	**F5**		**F6**	
	**f1**	**f2**	**f1**	**f2**
pH 6,8	6	44	30	26
Apple juice	14	38	15	42
Chamomile tea	18	30	37	23

*Profiles are similar if f1 < 15 and f2 is between 50 and 100. Profiles between F5 and F6 are differenced in all three mediums (f1 = 33, 25, 46, and f2 = 26, 29, 17 in pH 6.8, apple juice and chamomile tea respectively)
